# Sex-biased topography effects on butterfly dispersal

**DOI:** 10.1186/s40462-020-00234-6

**Published:** 2020-12-14

**Authors:** Elisa Plazio, Terezie Bubová, Vladimír Vrabec, Piotr Nowicki

**Affiliations:** 1 Institute of Environmental Sciences, Jagiellonian University, Gronostajowa 7, 30–387 Kraków, Poland; 2 Department of Zoology and Fisheries, Czech University of Life Sciences, Kamýcká 129, Suchdol, 165 21 Prague 6, Czech Republic

**Keywords:** Habitat fragmentation, Landscape, *Maculinea* (*Phengaris*), Mating strategies, Metapopulation

## Abstract

**Background:**

Metapopulation persistence in fragmented landscapes is assured by dispersal of individuals between local populations. In this scenario the landscape topography, although usually neglected, may have an important role in shaping dispersal throughout the matrix separating habitat patches. Due to inter-sexual differences in optimal reproductive strategies, i.e., males maximizing the number of mating opportunities and females maximizing the offspring survival chances, topography-related constraints are expected to exert a different effect on male and female dispersal behaviour. We tested sex-biased topography effects on butterfly dispersal, with the following hypotheses: (1) females are constrained by topography in their movements and avoid hill crossing; (2) male dispersal is primarily driven by two-dimensional spatial structure of the habitat patches (i.e. their geometric locations and sizes) and little influenced by topography.

**Methods:**

Following intensive mark-recapture surveys of *Maculinea* (= *Phengaris*) *nausithous* and *M. teleius* within a landscape characterised by an alternation of hills and valleys, we investigated sex-specific patterns in their inter-patch movement probabilities derived with a multi-state recapture model. In particular, we (1) analysed the fit of dispersal kernels based on Euclidean (= straight line) vs. topography-based (= through valley) distances; (2) compared movement probabilities for the pairs of patches separated or not by topographic barriers; and (3) tested the differences in the downward and upward movement probabilities within the pairs of patches.

**Results:**

Euclidean distances between patches proved to be a substantially stronger predictor of inter-patch movement probabilities in males, while inter-patch distances measured along valleys performed much better for females, indicating that the latter tend to predominantly follow valleys when dispersing. In addition, there were significantly lower probabilities of movements across hills in females, but not in males.

**Conclusions:**

Both above results provide support for the hypothesis that topography restricts dispersal in females, but not in males. Since the two sexes contribute differently to metapopulation functioning, i.e., only female dispersal can result in successful (re)colonisations of vacant patches, the topography effects exerted on females should be considered with particular attention when landscape management and conservation actions are designed in order to maintain the functional connectivity of metapopulation systems.

## Background

Dispersal is one of the key life-history traits which allow species to persist in fragmented landscapes. The proximate factors that drive an individual to actively move from its natal habitat patch vary but include local population conditions such as conspecific density, resource availability, stochastic environmental events, e.g., weather, habitat disturbance [45]. Ultimately, dispersal may help to avoid inbreeding and reduce competition for resources and mates, thereby increasing individual fitness [39]. Despite the benefits, the process of dispersal also induces potential costs that dispersing individuals may have to pay [9]. First, there is a relatively high mortality risk during the movement from natal sites to new areas mainly due to the lack of resources or unfavourable conditions in the matrix separating habitat patches [30, 86]. Apart from this, the metabolic costs due to increased energy expenditure during the transfer should be considered. Moreover, having reached a target site, dispersers may suffer reduced survival or lower reproductive success because of difficulties in resettling in a novel environment (e.g. [15, 46]).

Analysing intra-specific variation in dispersal abilities is especially important in the case of endangered species occurring in highly fragmented landscapes. However, dispersal studies in the past often assumed dispersal abilities to be fixed species-specific traits, especially in the classic formulation of the metapopulation theory [11, 35, 91], where individual movements between populations are modelled as a function of the fixed dispersal parameters of the focal species on the one hand and of the extremely variable spatial structure of their habitat patches on the other hand. Only recently there have been a number of empirical studies conducted on various animal taxa, from invertebrates to large mammals, which documented intra-specific variability in dispersal traits in response to various drivers acting as selective pressures on dispersal abilities. Many studies have documented the effect of patch system structure, i.e., size, quality, and connectivity of habitat patches, on emigration rates and dispersal distances, where, in general, large and high-quality patches separated by shorter distances favor higher dispersal rates and longer dispersal distances (e.g. [4, 20, 29, 79]). Likewise, higher dispersal rates and longer movement distances have been documented as a result of permeable landscape, constituted by the presence of corridors or stepping stones within the hostile landscape matrix (e.g. [30, 58, 66]). In addition, structural similarity between the landscape within the matrix and in the habitat patches is known to exert a positive effect on emigration frequency [30]. Finally, dispersal between natal and target sites is also shaped by the influence of landscape barriers on animal movements and individual propensity to cross such barriers [23].

Among other flying insects, butterflies have long been regarded as convenient models for dispersal studies due to their occurrence in discrete local populations that form metapopulation systems as well as to their ability to fly through an inhospitable matrix [40, 43]. On the other hand, certain landscape features may impede butterfly movements. It is worthwhile to stress that the perception of what constitutes a barrier for butterfly dispersal is not universal [47, 98]. For instance, some butterfly species avoid crossing water bodies (e.g. [98]), while others do not cross forests or tree plantations while dispersing between their habitat patches (e.g. [47, 75]). Moreover, a number of studies have pointed out inter- and intra-specific differences in propensity of butterflies to cross barriers, influenced by their age, sex, and morphological or ecological traits discriminating between good or poor flyers (e.g. [47, 52, 80]). Nevertheless, the aforementioned studies focused on landscape barriers and rarely considered the effects of topography on butterfly movements, despite the fact that topographic barriers are likely to play an important role as well, e.g., by influencing the perceptual range of moving individuals [69]. Besides, topography changes spatial distances, e.g., a movement over or around a hill separating habitat patches becomes longer. Finally, variation in topography may also be associated with changes in local vegetation [31], and in such a case dispersal is influenced by topography and matrix composition acting synergistically.

In fact, three-dimensional topography should be considered as a major factor capable of influencing dispersal patterns through directing animal movements into particular pathways, as revealed by some recent studies into the movements of large mobile organisms such as birds or mammals (e.g. [1, 10, 36]). Similar studies in small animals like insects have so far been very rare and restricted to hilltopping butterflies [67, 68, 70–72] and moths [37], characterised by a peculiar mate finding strategy in which males and virgin females ascend hills to mate. Nevertheless, three-dimensional topography may be expected to affect movements in any butterfly species.

More importantly, its effects are likely to be different for male and female dispersal behaviour. The latter prediction derives from the physiological differences of males and females, which limit female dispersal due to their substantial resource investment in producing eggs (cf. ‘oogenesis-flight syndrome’, [59]). This differential reproductive investment may affect dispersal propensity of both sexes in two ways. First, it leads to inter-sexual differences in optimal reproductive strategies [77], i.e., males aim to maximize the number of mating opportunities and females maximize the offspring survival chances. In this scenario, males of non-territorial species are more likely to disperse to increase their access to new females (e.g. [22]), while females are more likely to disperse when oviposition sites are scarce or poor quality (e.g. [3]). Second, the differential reproductive investment may also trigger morphological differences between sexes.

In many butterfly species females are bigger and have greater wings than males [56]. This characteristic may, in some cases, favour their movement abilities in a two-dimensional space, allowing them to have higher emigration rates, longer movement distances, and higher probability of crossing barriers of different nature (e.g. [47, 62, 83]). On the other hand, having greater wings is not necessarily a helpful trait in terms of flight performance, which also depends on other morphological characteristics. For instance, thorax mass has been proven to enhance flight performance (e.g. [87]), flight muscle ratio in the thorax enhances acceleration capacity [27], and the position of the centre of body mass directly relates to manoeuvrability [84]. These features may influence female flight performance, making them poorer flyers in the case of highly energy-demanding active flight (e.g. [17, 48, 51]), especially needed for crossing a topographic barrier, like a hill, which requires an active upward flight.

Furthermore, a strong evolutionary pressure is exerted on female dispersal behaviour, because female fitness is dependent on the ability to disperse safely and lay eggs in an appropriate habitat so as to assure the offspring survival. Females should normally move from a foodplant to nearby foodplant in order to lay eggs, but if foodplants are scarce, they should activate their ability to disperse in order to find another habitat patch [74]. Nevertheless, the interaction between genetically determined movement abilities and proximate stimuli from the external environment which trigger dispersal behaviour is modulated according to a cost/benefit balance. Hence, risky dispersal behaviour in females may be selected against, and thus females should be less prone (or less able) to cross topographic barriers (e.g. hills) when dispersing, being in turn constrained to follow easier and safer pathways (e.g. valleys). This is especially likely in the case of wet meadow butterflies as their crucial resources, such as nectar plants or larval foodplants, are more often found along valley beds.

Males, instead, appear to be less subjected to similar limitations. The strategy to maximise the number of encounters with receptive females should presumably make them more prone to undertake more risk while dispersing. In terms of cost-benefit balance, the evolutionary advantage of leaving a natal patch in search of another one in the case of female scarcity or high male density in order to decrease male-male competition for mating [74], could favour male fitness more than avoiding risky or more energy-demanding dispersal. Moreover, classic literature highlights that males of the species undertaking patrolling as a mate searching strategy have to move substantially more than territorial species that strictly adopt a sit-and-wait strategy [28]. Patrolling males fly slowly but continuously through the habitat in order to look for mates, targeting and finally approaching any insect having similar coloration and size to conspecific females [81]. Within-patch mate searching strategies in males of different butterfly may indirectly also affect their rates of dispersal, such that genuine perchers (adopting a sit-and-wait strategy) disperse less, where males of the species adopting patrolling behaviour tend to be better adapted to inter-patch dispersal (e.g. [5, 18, 95]). Such patrolling strategies are relatively common among butterflies, resulting in, on average, higher dispersal rate or longer distances travelled by males (e.g. [16, 33, 48]). Besides, the ability of males of some species to perform vertical spiral flights to deter other males [21] or to move above forests surrounding their meadow habitats [5, 21] may be an indication of their better adaptation to perform energetically demanding ascension flight which is required to cross topographic barriers.

Relaying on the above rationale, the purpose of the present study was to analyse how topography influences dispersal of males and females within metapopulations of the specialist butterflies *Maculinea* (= *Phengaris*) *nausithous* and *M. teleius*, occurring sympatrically in a complex landscape of valleys and hills. Due to inter-sexual differences in optimal reproductive strategies added to morphological and physiological differences in the two sexes, topography is expected to exert a different effect on male and female dispersal behaviour. We tested the following hypotheses: (1) females are constrained by topography in their movements and avoid hill crossing; (2) male dispersal is primarily driven by two-dimensional spatial structure of the habitat patches (i.e., their geometric locations and sizes) and little influenced by topography.

## Methods

### Study species

*M. nausithous* and *M. teleius* are globally classified as Near Threatened and Vulnerable, respectively [94], and they represent flagship species for conservation of grassland biodiversity in Europe [89]. Both are associated with wet meadows and are well known for their highly specialised life cycle, requiring two essential resources, namely *Sanguisorba officinalis* foodplants, which constitute primary nectar sources for adults as well as the exclusive initial larval food, and specific host ants of the genus *Myrmica*, in the colonies of which *Maculinea* larvae complete their development acting as social parasites [88]. These butterflies live sympatrically on the wet meadows. Host ants are typically wide-spread but scarce, thus representing the limiting factor for local abundances of *Maculinea* butterflies, while foodplants typically grow in high densities but are patchily distributed and, hence, they define the spatial extent of *Maculinea* habitat patches [2, 62]. Consequently, *M. nausithous* and *M. teleius* often occur sympatrically and form classic metapopulation systems with discrete local populations of relatively small size usually reaching several tens to several hundred adults [25, 64]. The flight period of both investigated species lasts roughly from the early July to mid-August. Adult butterflies are relatively sedentary, and only a few percent of individuals emigrate from their natal habitat patches, with typical dispersal distances in the range of a few hundred meters, and maximum movements reaching up to a few kilometres [7, 65, 74].

### Study area

**Fig. 1:**
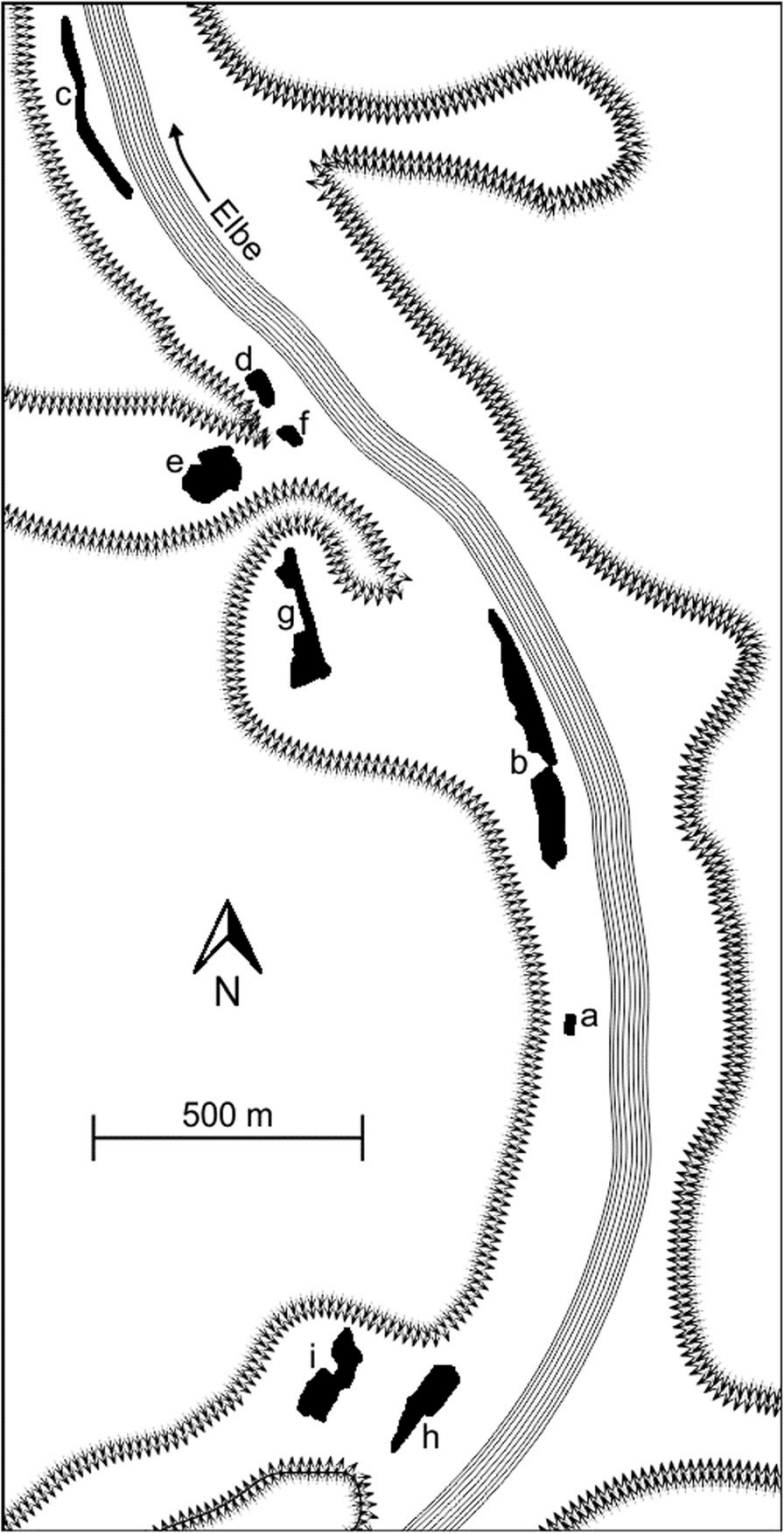
Map of the investigated habitat patches (a–i) in the Elbe river valley, with the arrow lines showing the slopes

The study area lies near the town of Dečin in the northern Czech Republic (50°49′N, 14°13′E). It is located within the Protected Landscape Area (PLA) Labské pískovce (Elbe Sandstone Mountains) and, more specifically, within the National Nature Reserve (NNR) Kaňon Labe (Elbe Canyon). The geological structure of the entire region is composed of massive layers of cretaceous sandstones [19, 53]. The Protected Landscape Area is therefore characterised by a typical sandstone landscape relief, with the Elbe Canyon constituting the largest sandstone canyon in Europe. It forms a narrow valley, with the maximum depth of the valley reaching up to 300 m, surrounded by relatively steep slopes of a hilly plateau. The main valley is joined by a number of short side valleys of the Elbe tributaries with similar relief (Fig. 1). The region has a temperate climate with a mean annual temperature of 8.0 °C and the annual precipitation sum reaching ca. 800 mm.

Wet meadows with *Maculinea* habitat patches are located in the main valley bed as well as in bottom fragments of the side valleys at the altitude of ca. 120–185 m a.s.l. Altogether, 10 local *Maculinea* populations were defined in the area [65]; however, one of them, marginally situated > 2.5 km north of the nearest other population, was excluded from the analyses in the present study. This is because butterfly movements to and from this population occurred only exceptionally; thus, reducing the number of populations considered allowed a much better precision of inter-patch dispersal estimates (as the number of model parameters grows factorially with the number of patches) with hardly any loss of information due to disregarded recaptures. The sizes of the nine analysed patches range between 0.03 and 1.38 ha. Their inter-patch distances typically reach several hundred meters to above 1 km, and the patches are either connected by valley systems or separated by hills of ca. 40–80 m in relative height.

### Field sampling

Local populations of both focal *Maculinea* species have been continuously monitored with mark-recapture surveys since 2008. However, sampling effort varied greatly among years, and consequently we had to restrict the present study to the data collected in 2010 and 2014, which proved to be the only years when all the habitat patches were sampled with adequate intensity to allow comprehensive analyses of inter-patch movements. It is worth noting that the 2010 data sets for both species were also used by Nowicki et al. [65], but this previous research relied on the evaluation of general levels of dispersal across the entire metapopulation (and compared them with the results for other metapopulations), whereas in the present study we focus more deeply on the detailed patterns of inter-patch movements within the metapopulation and how they are affected by topography.

The mark-recapture surveys were conducted throughout the whole flight period duration, i.e., from 9 July to 16 August in 2010 and from 3 July to 18 August in 2014. All the habitat patches were visited between 9:00 and 17:00 on a daily basis, weather allowing. In total there were 29 sampling days in 2010 and 38 sampling days in 2014. To ensure adequate sampling effort, the time spent in each patch was adjusted to its area and the abundance of butterflies flying (based on our experience from earlier years), varying from 0.3 to 1.5 person-hours per day, which assured the typical daily capture probabilities of ca. 20–40% individuals per patch. Butterflies were captured with entomological nets and marked with a unique code, composed of a letter and numbers written on the underside of the left hind wing with soft-tipped permanent markers, which is a well-established marking technique for butterflies (e.g. [7, 51, 65, 74]). Afterwards the captured individuals were immediately released at the spot of capture. For each (re)capture event we recorded the individual code and sex as well as patch ID, day and exact time.

### Analysis

The mark-recapture data collected were analysed using the multi-state recapture model of Brownie et al. [12] in MARK 8.0 software [97]. The model provides estimates of capture probability (*p*) and survival (*φ*) within each state as well as the transition probability between states (*ψ*), although the first two parameters were not of interest for the present study. The habitat patches investigated were adopted as different states, and consequently the transition probabilities represented the probabilities of butterfly movements between the patches. With nine patches analysed, there were 36 different pairs of patches, and thus 72 possible movement directions.

Separately for each of the two species, we tested different model variants, assuming distinct patterns in their parameters, which included a constant value denoted as (.), sex effect (*s*), temporal variation (*t*), additive sex effect and temporal variation (*s* + *t*), and their interactive effects (s*t). Nevertheless, based on Akaike Information Criterion corrected (AIC_c_) for small sample size [44], the model with constant survival and capture probability as well as sex-dependent transition probabilities, i.e. *φ*(.)*p*(.)*ψ*(*s*), was clearly the best supported for both years of study (2010 and 2014) and two species surveyed (*M. nausithous* and *M. teleius*).

It must be stressed that the transition (= movement) probability estimates of the multi-state recapture models represent unconfounded variables, and were usually a priori regarded as independent from one another in dispersal studies using a similar approach [32, 54, 66, 76]. Nevertheless, their non-independence in some cases, e.g. among movements starting from particular habitat patches or between certain patches, cannot be fully excluded, and thus prior to the proper analyses of topography effects on movement probabilities, we conducted preliminary testing for their independence within each year, species, and sex. Potential similarity among the probabilities of movements starting from particular patches (patch-based classes) as well as among the probabilities of movements along particular routes (in two directions) between pairs of patches (route-based classes) were assessed with intra-class correlation coefficients [49, 57]. In addition, we investigated possible spatial autocorrelations in estimated movement probabilities. For this purpose, the relationship between dissimilarity in movement probabilities and spatial distances separating the movements was tested with Mantel test based on 10,000 permutations [55]. Spatial distance between movements was calculated in two ways: either using their starting positions defined by the co-ordinates of the natal (= movement start) patch or using the movement centre position defined by the means of the natal and the target patch co-ordinates.

Since the outcome of all the aforementioned testing was clearly negative (see the Results section), we used the derived estimates of inter-patch movement probabilities as input for subsequent statistical analyses. Non-parametric tests were applied as the distributions of inter-patch movement probabilities were clearly right-skewed and zero-inflated in all the cases. Dispersal rates, reflected by movement probabilities, across years, species, and sexes were compared using Kruskal-Wallis ANOVA. All further analyses, focused on topography effects on butterfly dispersal, were conducted separately for each year, species, and sex.

First, we evaluated whether the butterfly movement probabilities are exclusively affected by geometric spatial structure of the habitat patches, i.e., their location and areas, or whether topography plays an important role in this respect as well. Since the probability of a movement between a particular pair of patches may be expected to decrease with increasing distance between the patches [41], we assessed if this relationship is better predicted by the Euclidean (straight line) inter-patch distances (*ED*) or by the inter-patch distances accounting for topography, i.e., measured along the valley beds (*VD*). The valley distances thus represent alternative inter-patch routes that entirely circumvent any hills. In the initial step, we compared the performance of Euclidean distance vs. valley distance as the sole predictor of inter-patch movements of investigated butterflies by testing the fit of a negative exponential function (NEF) using both distances to the obtained values of movement probabilities:

1 $$ {\psi}_{\mathrm{ij}}=k\cdotp \exp \left(-{\alpha D}_{\mathrm{ij}}\right) $$

where *D*_*ij*_ is the distance between patches *i* and *j* reflected either by Euclidean distance or by valley distance, while *k* and *α* represent the NEF parameters. It should be noted that 1/*α* corresponds to average estimated movement distance [41].

We adopted a NEF as the distance-dependence function since it is commonly applied as the dispersal kernel in butterfly studies [41] and proved to describe very well the dispersal of *Maculinea* butterflies in earlier studies [7, 62]. Subsequently, we also tested the fit of more complex versions of distance-dependence functions:

2 $$ {\psi}_{\mathrm{ij}}=k\cdotp \exp \left(-{\alpha D}_{\mathrm{ij}}\right)\cdotp {N}_i^{\zeta } $$

and

3 $$ {\psi}_{\mathrm{ij}}=k\cdotp \exp \left(-{\alpha D}_{\mathrm{ij}}\right)\cdotp {N}_i^{\zeta}\cdotp {T}_j^{\xi } $$

where *N*_*i*_ and *T*_*j*_ represent the areas of the natal patch and the target patch, respectively, while ζ and ξ are the respective scaling parameters. This was done in order to account for the potential effects of the natal and target patch sizes on the inter-patch movement probability because the area of a given patch is likely to negatively affect emigration from the patch and it may be expected to positively influence the chances of immigration into the patch [38, 41].

The performance of the dispersal kernels relying on Euclidean distance vs. their alternatives using valley distance was assessed with the proportion of variance they explained (*R*^2^). We also conducted model selection based on AICc. The models with AICc differing from the minimal one by less than two (ΔAICc < 2) were regarded as supported, and the model with the smallest number of parameters from among the supported models was considered the most appropriate, following the principle of parsimony [13].

In addition, we evaluated whether the investigated species are constrained in their movements by topography in an alternative way. Assuming that the butterflies may primarily move throughout the valleys and avoid crossing topographic barriers, we compared the movement probabilities between the patches separated or not separated by a hill with the Mann-Whitney test. The pairs of patches were assumed to be separated by a hill if they were located in different valleys and moving between them in relatively straight line would require crossing a hill. The outcome of the above testing was apparently not influenced by the expected distance-dependence of movement frequencies because the inter-patch distances in both groups were very similar (patches not separated by a hill: range = 142–2504 m, median = 1035 m; patches separated by a hill: range = 150–2404 m, median = 1124 m; Mann-Whitney test: *Z* = 0.7153, *P* = 0.4744).

Finally, we analysed if the downward moves, which are supported by gravity force and should thus be less demanding energetically, were more common than upward moves. In this case, the probabilities of movements from a higher located patch to a lower one and in the opposite direction within each pair of patches were compared with the Wilcoxon matched pairs test. Since this test relies on ranking the differences between the paired values, the pairs of patches for which the movement probabilities were estimated at zero in both directions (and thus equal) had to be excluded from the analysis.

All the statistical tests were performed in Statistica 13.0 [85], apart from the Mantel tests, which were carried out in the Zt program [8].

## Results

Over 2 years of the study we recorded 1424 individuals of *M. nausithous*, which were captured 2570 times. The other species, *M. teleius*, was far less abundant in the area, and the respective numbers reached 508 individuals and 815 captures. In general, males were captured more frequently than females, which reflects the typical slightly higher catchability of the former sex (cf. [63, 90]). Detailed information of the sample sizes for all the groups analysed is presented in Additional file 1.

**Fig. 2:**
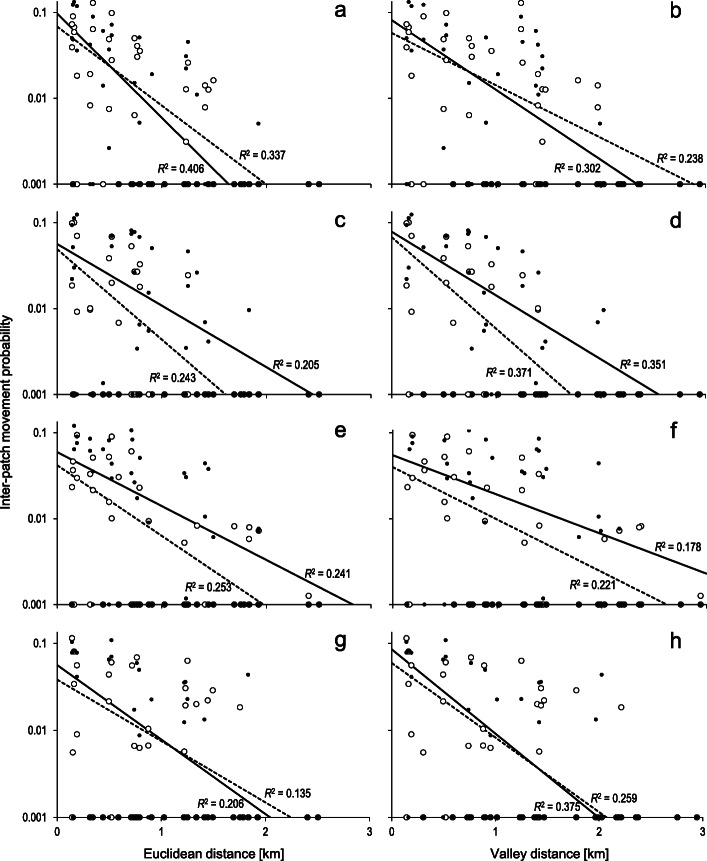
The inter-patch movement probabilities of *M. nausithous* (males (**a**, **b**); females (**c**, **d**)) and *M. teleius* (males (**e**, **f**); females (**g**, **h**)) in relation to inter-patch distances measured in a straight line (Euclidean distance) or along valleys (Valley distance). Note the logarithmic scale used, with zero values presented at 0.001. Empty dots depict the estimates for 2010, whereas solid dots show the 2014 estimates. The lines represent best-fit negative exponential functions (NEFs; broken line = 2010; solid line = 2014), accompanied with their proportions of variance explained (*R*^2^). Full details of the NEF fitting are given in Additional file 3

The inter-patch movement probabilities estimated with the multi-state recapture model ranged from 0 to slightly above 12%, although in most cases they were below 3%. The tests for non-independence of the movement probability estimates brought invariably insignificant results (Additional file 2). Intra-class correlation analysis revealed no consistency among movements from particular patches or along specific routes. Likewise, there was little indication of spatial autocorrelation in the movement probabilities, with the Mantel *r*_*M*_ values always close to 0. The levels of movement probabilities were highly similar in all the butterfly groups analysed (Fig. 2) and they did not depend on species, year, or sex (Kruskal-Wallis ANOVA: *H*_7, 576_ = 5.365; *P* = 0.6155; df = 7).

**Fig. 3:**
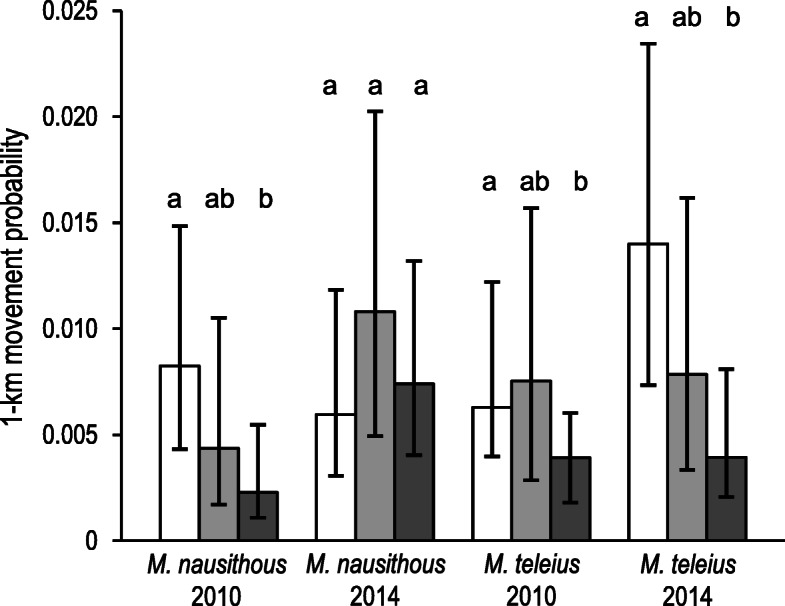
Probabilities of a movement to 1-km distant patch estimated for investigated *Maculinea* butterflies with the best-fit NEF dispersal kernels (white bars = males; light grey bars = females; dark grey bars = females following valleys). Different letters indicate significant differences (at *P* < 0.05) between groups within species and year

The NEF fitting revealed that the Euclidean distance between habitat patches performed much better than inter-patch distance measured along valleys as a predictor of male movement probabilities in both species and years (Fig. 2; Additional file 3). In females the pattern was exactly the opposite and the valley distance was invariably a better predictor of movement probabilities. The estimated average movement distances were in general slightly longer for males (*M. nausithous*: ca. 400–700 m; M. *teleius*: ca. 500–900 m) than in females (both species ca. 400–600 m), but the differences were far from significant for any species or year (see the *α* parameter values and their overlapping SEs in Additional file 3). Likewise, the probabilities of a movement to 1-km distant patch derived with the best-fit NEF dispersal kernels were not significantly different between males and females. However, when the topography impact was accounted for, i.e. the movements along valleys were assumed in females, their 1-km movement probabilities turned out substantially (ca. 2–3 times) lower than those of males, except for *M. nausithous* in 2014 (Fig. 3).

Inclusion of natal patch area and target patch area in the movement kernel typically did not bring a considerable improvement in the function fit (Additional file 3), but more importantly the general pattern remained the same, i.e., the models including Euclidean distance always performed better in males, while those relying on valley distance did so in females. The above results were confirmed by the outcome of the model selection based on the Akaike Information Criterion corrected for small sample size (Additional file 4). The models relying on Euclidean inter-patch distance consistently performed better for males, while their alternatives using the valley distance performed better for females. Similarly, including natal and/or target patch area in the models very rarely enhanced their performance.

**Fig. 4:**
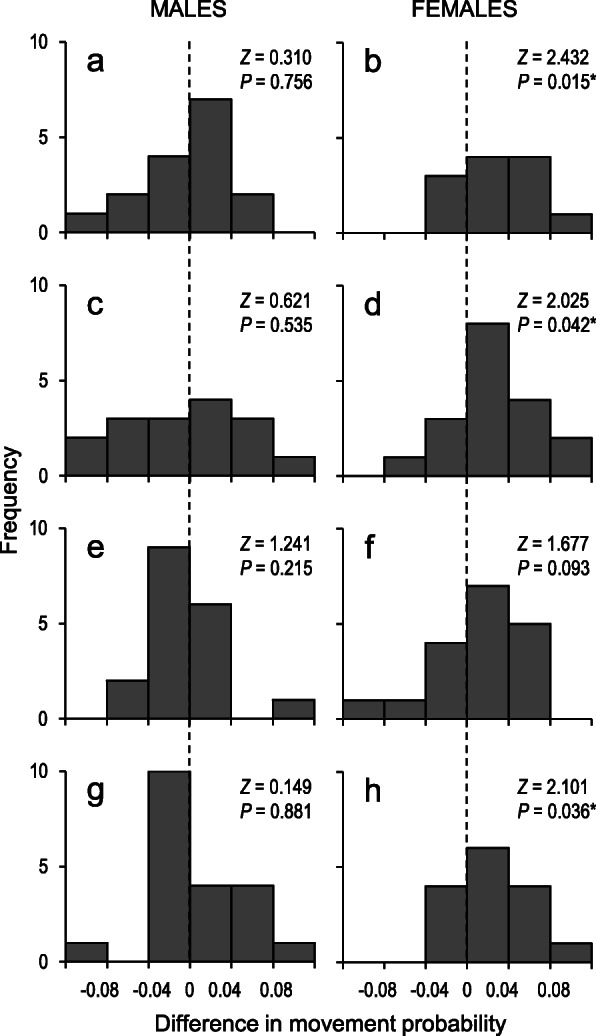
Distributions of the differences between downward and upward movement probabilities of *M. nausithous* (2010 (**a**, **b**); 2014 (**c**, **d**)) and *M. teleius* (2010 (**e**, **f**); 2014 (**g**, **h**)) within pairs of habitat patches. The results of the Wilcoxon matched pairs tests are presented in each case, with significant values (at *P* < 0.05) marked with asterisks. The broken lines indicate zero difference points, and thus the bars on the right of the lines represent the cases where downward movements prevailed, while those on the left refer to the upward movement prevalence cases

In concordance with the above kernel fitting outcomes, we found that female movement probabilities were significantly lower between patches separated by hills in all the cases except *M. teleius* in 2014, for which the difference approached the statistical significance level (Table 1). In turn, in male butterflies the movement probabilities did not differ significantly for the pairs of patches separated, or not, by hills. Similarly, downward movements decisively prevailed in females, except for a nearly significant case of *M. teleius* in 2010, whereas in males no significant differences could be detected, as downward and upward movement probabilities were fairly balanced (Fig. 4).

**Table 1:** *Maculinea* movement probabilities between habitat patches separated or not by topographic barriers (= hills)

Species	Year	Sex	Mann-Whitney test				
			Sum of ranks		*U*	*Z*	*P*
			no barrier (*n* = 36)	barrier (*n* = 36)			
*M. nausithous*	2010	males	1403.5	1224.5	558.5	1.1658	0.2437
		females	1489	1139	473	2.6396	0.0083^**^
	2014	males	1330	1298	632	0.2109	0.8329
		females	1555.5	1072.5	406.5	3.1567	0.0016^**^
*M. teleius*	2010	males	1407	1221	555	1.2588	0.2081
		females	1469	1159	493	2.1026	0.0355^*^
	2014	males	1408	1220	554	1.2111	0.2258
		females	1437	1191	525	1.7793	0.0752

The results of the Mann-Whitney tests comparing *Maculinea* movement probabilities between habitat patches separated or not separated by a topographic barrier are shown. Significant values are marked with asterisks (^*^ *P* < 0.05; ^**^ *P* < 0.01)

## Discussion

When analysing dispersal processes in metapopulations it is necessary to consider not only the structural connectivity of the local populations, defined by the locations and sizes of their habitat patches, but also the characteristics of the landscape, especially the composition of habitat types within matrix [42]. Indeed, many empirical studies demonstrated the cases of either positive or negative effects of specific features of matrix separating habitat patches (see review in [86]), also analysing their differential effect on male and female butterflies. In particular, [86] pointed out that the landscape permeability to butterfly dispersal primarily depends on whether environments forming the matrix are structurally similar to the habitats of a species or clearly distinct from them. In the case of grassland butterflies, major differences may be expected between dispersal in inhospitable matrixes like forests, structurally and qualitatively different from the habitat, and hospitable open environments including meadows, fallow lands, agricultural fields, road margins or even low-density residential areas, structurally similar to the habitat [65].

Previous studies into the impact of landscape structure and permeability on dispersal propensity in *Maculinea* butterflies often highlighted the ability of females to disperse more efficiently than males (e.g. [50, 62, 83]). Nevertheless, virtually all the studies concerning the effects of matrix on butterfly dispersal (including the aforementioned studies on *Maculinea*) considered landscape as a two-dimensional space in which individuals move. On the contrary, natural environments make three-dimensional systems, and even in the case of flying animals the landscape topography is likely to impede dispersal in certain directions, as vertical movements may be limited by physical and/or energetic constraints that may be different in the two sexes. Specifically, in butterflies the ability to perform vertical flights is different in males and females [5], which may thus be expected to react dissimilarly to the topographic barriers while dispersing.

In agreement with the above prediction, the findings of the present study reveal strong differences in the way *M. nausithous* and *M. teleius* adults of both sexes are influenced by topography. Our results showed that while males and females disperse with similar frequencies, the former are fairly independent from topography in their movements. In turn, females tend to follow easier routes along valleys as suggested by the substantially better fit of the dispersal kernels using the valley distances. This outcome, consistent for both *M. nausithous* and *M. teleius* and in the 2 years of our study, was further supported by the significantly lower female movement probabilities between habitat patches separated by a hill. Apart from being presumably less energetically demanding (as discussed in the Introduction), movements along valleys offer better chances of finding other patches of suitable habitat, since the *S. officinalis* foodplants grow in wet meadows occupying valley beds. The above explanation for female propensity to stick to the valley routes is in line with the findings of Schultz et al. [80], who demonstrated that female butterflies prefer to move through the areas where crucial resources, such as nectar plants or larval foodplants, are more likely to be found.

In contrast, male movement probabilities were predominantly affected by the geometric spatial structure of the habitat patches, as implied by better fit of dispersal kernel relying on Euclidean inter-patch distances, and no significant differences were found when comparing movement probabilities for pairs of habitat patches separated by a hill or not. The presence of topographic barriers therefore does not appear to constitute a major obstacle for male movements. Several earlier studies (e.g. [47, 80]) found that males are willing to cross habitat barriers (such as forests in the case of grassland butterflies) if this allows them to reach other fragments of suitable habitat in an efficient way. Additionally, males were found to be less sensitive than females to the effect of isolation of their metapopulation system, thus experiencing under such conditions less decrease in their movement distances than females [7]. Besides, in the case of hilltopping species, males actually take advantage of ascending hills which are used as mating spots [67, 68, 71, 72]. Even though hilltopping behaviour has never been described in *Maculinea* butterflies, the typical patrolling activity of males can make them more likely to reach higher elevations, as long as the hill vegetation allows to be penetrated with routine within-habitat movements. In fact, a hill may constitute an optimal point of observation to detect females in the surrounding area.

Analysing the probabilities of upward and downward movements within pairs of habitat patches, we found no particular tendency in males, while females were clearly more prone to move towards lower lying patches. This dissimilarity in the dispersal behaviour of the two sexes can possibly be once again attributed to the inter-sexual differences in morphology which, in turn are ultimately shaped by natural selection. The ‘oogenesis-flight syndrome’ postulate (cf. [59]) gives an evolutionary explanation to the possible limits in female dispersal, as individuals of this sex gain substantial fitness advantage when investing more resources in egg production. Due to the weight of the eggs they carry, butterfly females are on average heavier than males in many species (e.g. [18, 48, 51]), including *Maculinea* [83]. Greater body weight may represent a constraint in pursuing prolonged active flights against the gravity force while dispersing [34]. Therefore, females may instead prefer to perform more passive downward flights, which require lower energy investment and can be supported by wind flow, especially considering they possess longer wings compared to males [83]. Although having longer wings represents an advantage which helps females to move longer distances and cross habitat barriers such as forests (e.g. [47, 83]), the same characteristic may not be efficient enough, or even represent an impediment, in the case of ascension flights, which are required for crossing topographic barriers such as hills. In our study area characterized by topographic barriers we found that females have lower probability to reach distant habitat patches when topography is accounted for.

Conversely, butterfly males perform movements primarily in order to find more (or better) mating partners [24], and they may be evolutionary advantaged by investing more energy in active flight and dispersing in all directions. Such a movement strategy in males is supported by their larger thoraxes, 90% of which are made of wing moving muscles [26, 60]. Although, in *Maculinea,* thorax width was found to have no influence on the movement distances travelled within habitat patches [83], a more developed thorax should still give males more power and directional control of their flights in the three-dimensional space, therefore also favouring a stronger vertical propulsion.

Such an explanation is supported by recent literature into co-evolution between wing morphology and flight behaviour. In particular, a review by Le Roy et al. [56], who analysed flight and wing shape across a wide spectrum of butterfly species, confirmed that wing length (specifically, wing length relative to the mean wing width) has a fundamental role in enhancing the ability to glide with the help of wind currents. On the other hand, shorter wings are more efficient in performing complex manoeuvres, therefore they are likely to have evolved as an adaptation for increasing the efficiency of flight in three dimensions in species characterized by zig-zag flights. Similarly, a larger thorax is typical for species capable of performing rapid take-off and high acceleration. Following this rationale, the difference in wing length and thorax size between males and females of *Maculinea* (cf. [83]) may result from an adaptation to different flight behaviours deriving from different mating strategies of the two sexes (i.e., dispersal undertaken to find mates or for oviposition). In particular, according to Van Dyck and Regniers [93] freshly emerged *Maculinea* females are ready to mate soon after their eclosure. It is thus reasonable to assume that emigrating females undertake post-mating dispersal in order to find proper place to lay eggs.

Previous studies into intra-specific variability in butterfly dispersal considered habitat-related differences and explained with the fact that butterflies moving throughout inhospitable matrix tend to fly continuously and follow relatively straight paths, rather than perform short and tortuous flights as they do within their habitat patches or similar environments (e.g. [52, 65, 78]). However, the aforementioned studies (including [65] who dealt with the same metapopulations as those investigated in the present study) only considered general movement patterns within entire metapopulations. Conversely, considering male and female dispersal strategies separately may lead to different conclusions.

Specifically, while the results of Nowicki et al. [65] suggested that *Maculinea* butterflies are able to efficiently cross the forest matrix surrounding their habitat patches, the present study, dealing with inter-sexual differences as well as the influence of topography on dispersal, makes it clear that only males are able to move relatively freely in all directions. In turn, females are likely to follow valleys, and hence their movements are affected by the topography of the landscape. It is noteworthy that, since only females are able to successfully colonise vacant habitat patches, the functional demographic connectivity of the entire system would ultimately depend on how females move through the landscape.

## Conclusions

To summarize, the results of our study provide support for the hypotheses that topography affects dispersal in butterflies. More interestingly, in the present study, the same topography proved to differently shape dispersal in the two sexes of the focal species. It is evident that intersexual differences in dispersal have serious consequences for maintaining the functional connectivity of local populations, thus being crucial for the functioning of entire metapopulation systems. This is because only post-mating female dispersal can result in successful colonisations leading to the reestablishment of populations at the patches that have experienced local extinctions [6]. Consequently, dispersing females may achieve high evolutionary success as long as they are able to safely reach suitable habitats and lay eggs there. For a female willing to undertake dispersal its direct energy investment costs as well as indirect costs of dispersal, related to the uncertainty of finding good quality and sufficiently abundant foodplants in a new habitat patch, are presumably lower in the case of movements along valleys than those crossing hills. For this reason, a strong evolutionary pressure may be expected to promote ‘safe’ dispersal mode and counter risky dispersal. In contrast, the evolutionary success of males depends on their ability to find mating partners in an efficient way. The resulting more explorative movement behaviour of males, which apparently makes them more prone to take the risk of crossing barriers, contributes more strongly to gene flow [73].

Complementing our results with the actual dispersal routes followed by butterflies obtained through individual tracking (cf. [96]) might help to further clarify the differential effect of topography on males and females butterflies already detected in the present study. This would be useful to set a specific conservation plan for the entire metapopulation. More generally, while keeping in mind the need to ensure the functional connectivity of entire metapopulation systems (see [14, 82, 92]), the topography effects should always be considered. Many landscape management plans already provide recommendations for improving matrix permeability by creating ecological corridors or setting aside small, otherwise undesired, fragments of land to act as stepping stones linking habitat patches [23, 61]. In addition to this practice, we highlight the necessity to consider also topography impacts on dispersal and potential intersexual differences in this respect. Specifically, in a situation like the one highlighted by our study, in order to ensure the functional metapopulation connectivity the management plan should account for topography-related constraints in female dispersal. In particular, corridors or stepping stones should optimally follow the valleys to support female movements, whereas establishing them on hills would be far less effective.

## Supplementary Information

**Additional file 1.** Results - Sample sizes of butterflies captured throughmark-capture surveys in the Elbe river valley near Dečin, northern CzechRepublic.

**Additional file 2. **Results - Testing of independence of movement probability estimatesderived for investigated *Maculinea*butterflies with the multi-state recapture model.

**Additional file 3.** Results - Results of the negative exponential function (NEF) fitting performed toevaluate the effects of inter-patch Euclidean distances (*ED*) vs. topographic distances measured along the valley beds (*VD*) as well as of natal (= movementstart) patch areas (*N*) and targetpatch areas (*T*) on the movementprobabilities (*ψ*) of investigated *Maculinea* butterflies.

**Additional file 4.** Results - Model selection based on the Akaike InformationCriterion corrected for small sample size.

## Data Availability

The datasets used and/or analysed during the current study are available from the corresponding author on reasonable request.

## Abbreviations

AICc: Akaike Information Criterion corrected
ANOVA: Analysis of Variance
ED: Euclidean distance
ID: Identification
NEF: Negative exponential function
NNR: National Nature Reserve
PLA: Protected Landscape Area
VD: Valley distance
