# Supplementary material for: Sex-biased topography effects on butterfly dispersal
Source: Mov Ecol. 2020 Dec 14;8:50. doi: 10.1186/s40462-020-00234-6 (PMC7737334; doi:10.1186/s40462-020-00234-6)
Supplement: Supplementary file 1 — Additional file 1. Results - Sample sizes of butterflies captured throughmark-capture surveys in the Elbe river valley near Dečin, northern CzechRepublic. [file 40462_2020_234_MOESM1_ESM.docx]

**Plazio E*, Bubová T, Vrabec V, Nowicki N (2020). Sex-biased topography effects on butterfly dispersal**

* Corresponding author. Email: [elisa.plazio@doctoral.uj.edu.pl](mailto:elisa.plazio@doctoral.uj.edu.pl)

**Additional file 1**. Sample sizes of butterflies captured through mark-capture surveys in the Elbe river valley near Dečin, northern Czech Republic.

| Species | Year | Individuals | | |  | Captures | | |
| --- | --- | --- | --- | --- | --- | --- | --- | --- |
|  |  | males | females | total |  | males | females | total |
| *M. nausithous* | 2010 | 476 | 375 | 851 |  | 797 | 546 | 1343 |
|  | 2014 | 301 | 272 | 573 |  | 743 | 484 | 1227 |
| *M. teleius* | 2010 | 169 | 148 | 317 |  | 261 | 209 | 470 |
|  | 2014 | 95 | 96 | 191 |  | 195 | 150 | 345 |
