# Supplementary material for: Sex-biased topography effects on butterfly dispersal
Source: Mov Ecol. 2020 Dec 14;8:50. doi: 10.1186/s40462-020-00234-6 (PMC7737334; doi:10.1186/s40462-020-00234-6)
Supplement: Supplementary file 2 — Additional file 2. Results - Testing of independence of movement probability estimatesderived for investigated Maculineabutterflies with the multi-state recapture model. [file 40462_2020_234_MOESM2_ESM.docx]

**Plazio E*, Bubová T, Vrabec V, Nowicki N (2020). Sex-biased topography effects on butterfly dispersal**

* Corresponding author. Email: [elisa.plazio@doctoral.uj.edu.pl](mailto:elisa.plazio@doctoral.uj.edu.pl)

**Additional file 2**. Testing of independence of movement probability estimates derived for investigated *Maculinea* butterflies with the multi-state recapture model. Intra-class correlation was evaluated for the probabilities of movements from particular natal patches (patch-based) as well as along particular routes (in two directions) between pairs of patches (route-based), whereas spatial autocorrelation was assessed with Mantel tests relying on the distances between movement start positions or between movement centre positions – see the text for details.

| Species | Year | Sex | Intra-class correlation | | | | |  | Spatial autocorrelation | | | | |
| --- | --- | --- | --- | --- | --- | --- | --- | --- | --- | --- | --- | --- | --- |
|  |  |  | patch-based  (*df* = 7,8) | |  | route-based  (*df* = 1,35) | |  | start position  (matrix: 72 × 72) | |  | centre position  (matrix: 72 × 72) | |
|  |  |  | *r_I_* | *P* |  | *r_I_* | *P* |  | *r_M_* | *P* |  | *r_M_* | *P* |
| *M. nausithous* | 2010 | males | 0.0212 | 0.2299 |  | 0.4555 | 0.1308 |  | –0.0289 | 0.2224 |  | –0.0352 | 0.1895 |
| *M. nausithous* | 2010 | females | 0.0104 | 0.7247 |  | 0.1926 | 0.8553 |  | –0.0359 | 0.1686 |  | 0.0610 | 0.0707 |
| *M. nausithous* | 2014 | males | 0.0116 | 0.6542 |  | 0.2884 | 0.6087 |  | –0.0131 | 0.3782 |  | 0.0727 | 0.0534 |
| *M. nausithous* | 2014 | females | 0.0075 | 0.8672 |  | 0.3800 | 0.2940 |  | –0.0182 | 0.3330 |  | 0.0749 | 0.0625 |
| *M. teleius* | 2010 | males | 0.0044 | 0.9716 |  | 0.3774 | 0.3018 |  | –0.0021 | 0.4620 |  | 0.0764 | 0.0626 |
| *M. teleius* | 2010 | females | 0.0092 | 0.7872 |  | 0.2243 | 0.8411 |  | 0.0193 | 0.2944 |  | 0.0467 | 0.0672 |
| *M. teleius* | 2014 | males | 0.0140 | 0.5263 |  | 0.4431 | 0.1305 |  | –0.0631 | 0.0685 |  | 0.0136 | 0.3578 |
| *M. teleius* | 2014 | females | 0.0067 | 0.9011 |  | 0.4574 | 0.1339 |  | 0.0353 | 0.1825 |  | 0.0525 | 0.0732 |
