# Supplementary material for: Sex-biased topography effects on butterfly dispersal
Source: Mov Ecol. 2020 Dec 14;8:50. doi: 10.1186/s40462-020-00234-6 (PMC7737334; doi:10.1186/s40462-020-00234-6)
Supplement: Supplementary file 3 — Additional file 3. Results - Results of the negative exponential function (NEF) fitting performed toevaluate the effects of inter-patch Euclidean distances (ED) vs. topographic distances measured along the valley beds (VD) as well as of natal (= movementstart) patch areas (N) and targetpatch areas (T) on the movementprobabilities (ψ) of investigated Maculinea butterflies. [file 40462_2020_234_MOESM3_ESM.docx]

**Plazio E*, Bubová T, Vrabec V, Nowicki N (2020). Sex-biased topography effects on butterfly dispersal**

* Corresponding author. Email: [elisa.plazio@doctoral.uj.edu.pl](mailto:elisa.plazio@doctoral.uj.edu.pl)

**Additional file 3**. Results of the negative exponential function (NEF) fitting performed to evaluate the effects of inter-patch Euclidean distances (*ED*) vs. topographic distances measured along the valley beds (*VD*) as well as of natal (= movement start) patch areas (*N*) and target patch areas (*T*) on the movement probabilities (*ψ*) of investigated *Maculinea* butterflies.

| Function | Constant | | |  | Inter-patch distance dependence | | |  | Natal patch size scaling | | |  | Target patch size scaling | | |  | *R*^2^ |
| --- | --- | --- | --- | --- | --- | --- | --- | --- | --- | --- | --- | --- | --- | --- | --- | --- | --- |
|  | *k* ± SE | *t* | *P* |  | *α* ± SE | *t* | *P* |  | *ζ* ± SE | *t* | *P* |  | *ξ* ± SE | *t* | *P* |  |  |
| ***M. nausithous*, 2010, males** | | | | | | | | | | | | | | | | | |
| $\psi_{ij}=k\cdot\text{exp(}-\alpha ED_{ij}\text{)}$ | 0.068 ± 0.014 | 4.835 | 0.0000 |  | 2.116 ± 0.546 | 3.873 | 0.0002 |  | --- | --- | --- |  | --- | --- | --- |  | 0.337 |
| $\psi_{ij}=k\cdot\text{exp(}-\alpha VD_{ij}\text{)}$ | 0.058 ± 0.013 | 4.376 | < 0.0001 |  | 1.404 ± 0.399 | 3.518 | 0.0008 |  | --- | --- | --- |  | --- | --- | --- |  | 0.238 |
| $\psi_{ij}=k\cdot\text{exp(}-\alpha ED_{ij}\text{)}\cdot N_{i}^{\zeta}$ | 0.037 ± 0.009 | 4.210 | 0.0001 |  | 2.179 ± 0.388 | 5.622 | 0.0000 |  | –0.435 ± 0.088 | –4.931 | < 0.0001 |  | --- | --- | --- |  | 0.520 |
| $\psi_{ij}=k\cdot\text{exp(}-\alpha VD_{ij}\text{)}\cdot N_{i}^{\zeta}$ | 0.036 ± 0.011 | 3.412 | 0.0011 |  | 1.467 ± 0.346 | 4.237 | < 0.0001 |  | –0.332 ± 0.105 | –3.154 | 0.0024 |  | --- | --- | --- |  | 0.339 |
| $\psi_{ij}=k\cdot\text{exp(}-\alpha ED_{ij}\text{)}\cdot N_{i}^{\zeta}\cdot T_{j}^{\xi}$ | 0.039 ± 0.012 | 3.278 | 0.0016 |  | 2.187 ± 0.396 | 5.517 | 0.0000 |  | –0.432 ± 0.096 | –4.505 | 0.0000 |  | 0.055 ± 0.124 | 0.440 | 0.6614 |  | 0.521 |
| $\psi_{ij}=k\cdot\text{exp(}-\alpha VD_{ij}\text{)}\cdot N_{i}^{\zeta}\cdot T_{j}^{\xi}$ | 0.041 ± 0.015 | 2.753 | 0.0076 |  | 1.445 ± 0.353 | 4.092 | 0.0001 |  | –0.319 ± 0.112 | –2.839 | 0.0060 |  | 0.136 ± 0.151 | 0.902 | 0.3704 |  | 0.345 |
| ***M. nausithous*, 2010, females** | | | | | | | | | | | | | | | | | |
| $\psi_{ij}=k\cdot\text{exp(}-\alpha ED_{ij}\text{)}$ | 0.049 ± 0.013 | 3.641 | 0.0005 |  | 2.419 ± 0.809 | 2.992 | 0.0038 |  | --- | --- | --- |  | --- | --- | --- |  | 0.243 |
| $\psi_{ij}=k\cdot\text{exp(}-\alpha VD_{ij}\text{)}$ | 0.068 ± 0.014 | 4.755 | 0.0000 |  | 2.457 ± 0.610 | 4.029 | 0.0001 |  | --- | --- | --- |  | --- | --- | --- |  | 0.371 |
| $\psi_{ij}=k\cdot\text{exp(}-\alpha ED_{ij}\text{)}\cdot N_{i}^{\zeta}$ | 0.031 ± 0.010 | 2.962 | 0.0042 |  | 2.133 ± 0.649 | 3.288 | 0.0016 |  | –0.276 ± 0.128 | –2.150 | 0.0350 |  | --- | --- | --- |  | 0.275 |
| $\psi_{ij}=k\cdot\text{exp(}-\alpha VD_{ij}\text{)}\cdot N_{i}^{\zeta}$ | 0.049 ± 0.013 | 3.713 | 0.0004 |  | 2.159 ± 0.507 | 4.257 | 0.0001 |  | –0.172 ± 0.104 | –1.664 | 0.1007 |  | --- | --- | --- |  | 0.386 |
| $\psi_{ij}=k\cdot\text{exp(}-\alpha ED_{ij}\text{)}\cdot N_{i}^{\zeta}\cdot T_{j}^{\xi}$ | 0.019 ± 0.009 | 2.042 | 0.0451 |  | 2.373 ± 0.604 | 3.927 | 0.0002 |  | –0.384 ± 0.150 | –2.551 | 0.0130 |  | –0.319 ± 0.152 | –2.099 | 0.0395 |  | 0.324 |
| $\psi_{ij}=k\cdot\text{exp(}-\alpha VD_{ij}\text{)}\cdot N_{i}^{\zeta}\cdot T_{j}^{\xi}$ | 0.033 ± 0.013 | 2.529 | 0.0138 |  | 2.180 ± 0.471 | 4.630 | 0.0000 |  | –0.249 ± 0.121 | –2.063 | 0.0429 |  | –0.209 ± 0.122 | –1.712 | 0.0915 |  | 0.416 |

Table S2. *continued*

| Function | Constant | | |  | Inter–patch distance dependence | | |  | Natal patch size scaling | | |  | Target patch size scaling | | |  | *R*^2^ |
| --- | --- | --- | --- | --- | --- | --- | --- | --- | --- | --- | --- | --- | --- | --- | --- | --- | --- |
|  | *k* ± SE | *t* | *P* |  | *α* ± SE | *t* | *P* |  | *ζ* ± SE | *t* | *P* |  | *ξ* ± SE | *t* | *P* |  |  |
| ***M. nausithous*, 2014, males** | | | | | | | | | | | | | | | | | |
| $\psi_{ij}=k\cdot\text{exp(}-\alpha ED_{ij}\text{)}$ | 0.097 ± 0.019 | 4.972 | 0.0000 |  | 2.788 ± 0.664 | 4.197 | 0.0001 |  | --- | --- | --- |  | --- | --- | --- |  | 0.406 |
| $\psi_{ij}=k\cdot\text{exp(}-\alpha VD_{ij}\text{)}$ | 0.081 ± 0.017 | 4.718 | 0.0000 |  | 1.872 ± 0.476 | 3.932 | 0.0002 |  | --- | --- | --- |  | --- | --- | --- |  | 0.302 |
| $\psi_{ij}=k\cdot\text{exp(}-\alpha ED_{ij}\text{)}\cdot N_{i}^{\zeta}$ | 0.079 ± 0.019 | 4.107 | 0.0001 |  | 2.743 ± 0.625 | 4.386 | 0.0000 |  | –0.152 ± 0.095 | –1.601 | 0.1140 |  | --- | --- | --- |  | 0.427 |
| $\psi_{ij}=k\cdot\text{exp(}-\alpha VD_{ij}\text{)}\cdot N_{i}^{\zeta}$ | 0.076 ± 0.020 | 3.726 | 0.0004 |  | 1.862 ± 0.475 | 3.921 | 0.0002 |  | –0.050 ± 0.112 | –0.446 | 0.6570 |  | --- | --- | --- |  | 0.304 |
| $\psi_{ij}=k\cdot\text{exp(}-\alpha ED_{ij}\text{)}\cdot N_{i}^{\zeta}\cdot T_{j}^{\xi}$ | 0.223 ± 0.060 | 3.726 | 0.0004 |  | 4.007 ± 0.656 | 6.111 | 0.0000 |  | –0.101 ± 0.076 | –1.331 | 0.1878 |  | 0.939 ± 0.245 | 3.831 | 0.0003 |  | 0.598 |
| $\psi_{ij}=k\cdot\text{exp(}-\alpha VD_{ij}\text{)}\cdot N_{i}^{\zeta}\cdot T_{j}^{\xi}$ | 0.238 ± 0.084 | 2.817 | 0.0063 |  | 3.254 ± 0.722 | 4.507 | 0.0000 |  | 0.014 ± 0.096 | 0.147 | 0.8837 |  | 0.836 ± 0.280 | 2.984 | 0.0040 |  | 0.434 |
| ***M. nausithous*, 2014, females** | | | | | | | | | | | | | | | | | |
| $\psi_{ij}=k\cdot\text{exp(}-\alpha ED_{ij}\text{)}$ | 0.056 ± 0.014 | 3.959 | 0.0002 |  | 1.650 ± 0.548 | 3.012 | 0.0036 |  | --- | --- | --- |  | --- | --- | --- |  | 0.205 |
| $\psi_{ij}=k\cdot\text{exp(}-\alpha VD_{ij}\text{)}$ | 0.078 ± 0.015 | 5.199 | 0.0000 |  | 1.709 ± 0.398 | 4.295 | 0.0001 |  | --- | --- | --- |  | --- | --- | --- |  | 0.351 |
| $\psi_{ij}=k\cdot\text{exp(}-\alpha ED_{ij}\text{)}\cdot N_{i}^{\zeta}$ | 0.079 ± 0.024 | 3.348 | 0.0013 |  | 1.890 ± 0.603 | 3.135 | 0.0025 |  | 0.271 ± 0.192 | 1.411 | 0.1626 |  | --- | --- | --- |  | 0.228 |
| $\psi_{ij}=k\cdot\text{exp(}-\alpha VD_{ij}\text{)}\cdot N_{i}^{\zeta}$ | 0.118 ± 0.027 | 4.420 | 0.0000 |  | 2.006 ± 0.442 | 4.540 | 0.0000 |  | 0.299 ± 0.141 | 2.127 | 0.0370 |  | --- | --- | --- |  | 0.398 |
| $\psi_{ij}=k\cdot\text{exp(}-\alpha ED_{ij}\text{)}\cdot N_{i}^{\zeta}\cdot T_{j}^{\xi}$ | 0.044 ± 0.017 | 2.562 | 0.0126 |  | 2.223 ± 0.538 | 4.130 | 0.0001 |  | 0.206 ± 0.188 | 1.097 | 0.2767 |  | –0.416 ± 0.123 | –3.378 | 0.0012 |  | 0.359 |
| $\psi_{ij}=k\cdot\text{exp(}-\alpha VD_{ij}\text{)}\cdot N_{i}^{\zeta}\cdot T_{j}^{\xi}$ | 0.067 ± 0.020 | 3.328 | 0.0014 |  | 1.990 ± 0.368 | 5.407 | 0.0000 |  | 0.216 ± 0.134 | 1.612 | 0.1116 |  | –0.304 ± 0.092 | –3.324 | 0.0014 |  | 0.496 |

Table S2. *continued*

| Function | Constant | | |  | Inter–patch distance dependence | | |  | Natal patch size scaling | | |  | Target patch size scaling | | |  | *R*^2^ |
| --- | --- | --- | --- | --- | --- | --- | --- | --- | --- | --- | --- | --- | --- | --- | --- | --- | --- |
|  | *k* ± SE | *t* | *P* |  | *α* ± SE | *t* | *P* |  | *ζ* ± SE | *t* | *P* |  | *ξ* ± SE | *t* | *P* |  |  |
| ***M. teleius*, 2010, males** | | | | | | | | | | | | | | | | | |
| $\psi_{ij}=k\cdot\text{exp(}-\alpha ED_{ij}\text{)}$ | 0.042 ± 0.010 | 4.063 | 0.0001 |  | 1.893 ± 0.595 | 3.184 | 0.0022 |  | --- | --- | --- |  | --- | --- | --- |  | 0.253 |
| $\psi_{ij}=k\cdot\text{exp(}-\alpha VD_{ij}\text{)}$ | 0.040 ± 0.010 | 4.010 | 0.0001 |  | 1.406 ± 0.436 | 3.223 | 0.0019 |  | --- | --- | --- |  | --- | --- | --- |  | 0.221 |
| $\psi_{ij}=k\cdot\text{exp(}-\alpha ED_{ij}\text{)}\cdot N_{i}^{\zeta}$ | 0.042 ± 0.013 | 3.354 | 0.0013 |  | 1.897 ± 0.602 | 3.151 | 0.0024 |  | 0.005 ± 0.137 | 0.040 | 0.9685 |  | --- | --- | --- |  | 0.253 |
| $\psi_{ij}=k\cdot\text{exp(}-\alpha VD_{ij}\text{)}\cdot N_{i}^{\zeta}$ | 0.044 ± 0.013 | 3.330 | 0.0014 |  | 1.463 ± 0.454 | 3.225 | 0.0019 |  | 0.067 ± 0.141 | 0.476 | 0.6354 |  | --- | --- | --- |  | 0.224 |
| $\psi_{ij}=k\cdot\text{exp(}-\alpha ED_{ij}\text{)}\cdot N_{i}^{\zeta}\cdot T_{j}^{\xi}$ | 0.133 ± 0.051 | 2.598 | 0.0115 |  | 2.830 ± 0.741 | 3.821 | 0.0003 |  | 0.065 ± 0.109 | 0.593 | 0.5553 |  | 1.579 ± 0.457 | 3.455 | 0.0010 |  | 0.381 |
| $\psi_{ij}=k\cdot\text{exp(}-\alpha VD_{ij}\text{)}\cdot N_{i}^{\zeta}\cdot T_{j}^{\xi}$ | 0.154 ± 0.057 | 2.706 | 0.0086 |  | 2.480 ± 0.635 | 3.908 | 0.0002 |  | 0.219 ± 0.125 | 1.748 | 0.0850 |  | 1.011 ± 0.338 | 2.990 | 0.0039 |  | 0.357 |
| ***M. teleius*, 2010, females** | | | | | | | | | | | | | | | | | |
| $\psi_{ij}=k\cdot\text{exp(}-\alpha ED_{ij}\text{)}$ | 0.038 ± 0.011 | 3.374 | 0.0012 |  | 1.624 ± 0.635 | 2.558 | 0.0127 |  | --- | --- | --- |  | --- | --- | --- |  | 0.135 |
| $\psi_{ij}=k\cdot\text{exp(}-\alpha VD_{ij}\text{)}$ | 0.059 ± 0.014 | 4.332 | 0.0000 |  | 1.972 ± 0.544 | 3.625 | 0.0005 |  | --- | --- | --- |  | --- | --- | --- |  | 0.259 |
| $\psi_{ij}=k\cdot\text{exp(}-\alpha ED_{ij}\text{)}\cdot N_{i}^{\zeta}$ | 0.034 ± 0.012 | 2.799 | 0.0066 |  | 1.556 ± 0.613 | 2.538 | 0.0134 |  | –0.066 ± 0.153 | –0.431 | 0.6679 |  | --- | --- | --- |  | 0.136 |
| $\psi_{ij}=k\cdot\text{exp(}-\alpha VD_{ij}\text{)}\cdot N_{i}^{\zeta}$ | 0.064 ± 0.019 | 3.426 | 0.0010 |  | 2.061 ± 0.577 | 3.572 | 0.0007 |  | 0.034 ± 0.131 | 0.263 | 0.7933 |  | --- | --- | --- |  | 0.260 |
| $\psi_{ij}=k\cdot\text{exp(}-\alpha ED_{ij}\text{)}\cdot N_{i}^{\zeta}\cdot T_{j}^{\xi}$ | 0.033 ± 0.096 | 0.341 | 0.7344 |  | 14.16 ± 13.90 | 1.018 | 0.3121 |  | –0.002 ± 0.355 | –0.007 | 0.9947 |  | –1.124 ± 0.465 | –2.419 | 0.0182 |  | 0.304 |
| $\psi_{ij}=k\cdot\text{exp(}-\alpha VD_{ij}\text{)}\cdot N_{i}^{\zeta}\cdot T_{j}^{\xi}$ | 0.096 ± 0.121 | 0.792 | 0.4310 |  | 7.901 ± 4.839 | 1.633 | 0.1071 |  | 0.174 ± 0.226 | 0.768 | 0.4454 |  | –0.458 ± 0.217 | –2.111 | 0.0384 |  | 0.335 |

Table S2. *continued*

| Function | Constant | | |  | Inter–patch distance dependence | | |  | Natal patch size scaling | | |  | Target patch size scaling | | |  | *R*^2^ |
| --- | --- | --- | --- | --- | --- | --- | --- | --- | --- | --- | --- | --- | --- | --- | --- | --- | --- |
|  | *k* ± SE | *t* | *P* |  | *α* ± SE | *t* | *P* |  | *ζ* ± SE | *t* | *P* |  | *ξ* ± SE | *t* | *P* |  |  |
| ***M. teleius*, 2014, males** | | | | | | | | | | | | | | | | | |
| $\psi_{ij}=k\cdot\text{exp(}-\alpha ED_{ij}\text{)}$ | 0.059 ± 0.013 | 4.526 | 0.0000 |  | 1.445 ± 0.434 | 3.332 | 0.0014 |  | --- | --- | --- |  | --- | --- | --- |  | 0.241 |
| $\psi_{ij}=k\cdot\text{exp(}-\alpha VD_{ij}\text{)}$ | 0.055 ± 0.013 | 4.154 | 0.0001 |  | 1.058 ± 0.338 | 3.126 | 0.0026 |  | --- | --- | --- |  | --- | --- | --- |  | 0.178 |
| $\psi_{ij}=k\cdot\text{exp(}-\alpha ED_{ij}\text{)}\cdot N_{i}^{\zeta}$ | 0.072 ± 0.019 | 3.879 | 0.0002 |  | 1.538 ± 0.453 | 3.397 | 0.0011 |  | 0.148 ± 0.142 | 1.043 | 0.3008 |  | --- | --- | --- |  | 0.254 |
| $\psi_{ij}=k\cdot\text{exp(}-\alpha VD_{ij}\text{)}\cdot N_{i}^{\zeta}$ | 0.064 ± 0.018 | 3.594 | 0.0006 |  | 1.103 ± 0.347 | 3.179 | 0.0022 |  | 0.109 ± 0.137 | 0.796 | 0.4287 |  | --- | --- | --- |  | 0.186 |
| $\psi_{ij}=k\cdot\text{exp(}-\alpha ED_{ij}\text{)}\cdot N_{i}^{\zeta}\cdot T_{j}^{\xi}$ | 0.084 ± 0.026 | 3.213 | 0.0020 |  | 1.582 ± 0.463 | 3.419 | 0.0011 |  | 0.163 ± 0.143 | 1.137 | 0.2597 |  | 0.113 ± 0.133 | 0.852 | 0.3970 |  | 0.261 |
| $\psi_{ij}=k\cdot\text{exp(}-\alpha VD_{ij}\text{)}\cdot N_{i}^{\zeta}\cdot T_{j}^{\xi}$ | 0.070 ± 0.024 | 2.910 | 0.0049 |  | 1.112 ± 0.351 | 3.167 | 0.0023 |  | 0.118 ± 0.140 | 0.838 | 0.4050 |  | 0.063 ± 0.130 | 0.483 | 0.6305 |  | 0.189 |
| ***M. teleius*, 2014, females** | | | | | | | | | | | | | | | | | |
| $\psi_{ij}=k\cdot\text{exp(}-\alpha ED_{ij}\text{)}$ | 0.056 ± 0.015 | 3.815 | 0.0003 |  | 1.971 ± 0.654 | 3.015 | 0.0036 |  | --- | --- | --- |  | --- | --- | --- |  | 0.206 |
| $\psi_{ij}=k\cdot\text{exp(}-\alpha VD_{ij}\text{)}$ | 0.085 ± 0.016 | 5.187 | 0.0000 |  | 2.229 ± 0.510 | 4.373 | 0.0000 |  | --- | --- | --- |  | --- | --- | --- |  | 0.375 |
| $\psi_{ij}=k\cdot\text{exp(}-\alpha ED_{ij}\text{)}\cdot N_{i}^{\zeta}$ | 0.048 ± 0.015 | 3.106 | 0.0027 |  | 1.983 ± 0.643 | 3.082 | 0.0030 |  | –0.134 ± 0.131 | –1.020 | 0.3115 |  | --- | --- | --- |  | 0.215 |
| $\psi_{ij}=k\cdot\text{exp(}-\alpha VD_{ij}\text{)}\cdot N_{i}^{\zeta}$ | 0.083 ± 0.020 | 4.058 | 0.0001 |  | 2.210 ± 0.510 | 4.330 | 0.0000 |  | –0.021 ± 0.104 | –0.206 | 0.8372 |  | --- | --- | --- |  | 0.375 |
| $\psi_{ij}=k\cdot\text{exp(}-\alpha ED_{ij}\text{)}\cdot N_{i}^{\zeta}\cdot T_{j}^{\xi}$ | 0.024 ± 0.012 | 2.012 | 0.0482 |  | 2.453 ± 0.603 | 4.065 | 0.0001 |  | –0.289 ± 0.154 | –1.873 | 0.0653 |  | –0.445 ± 0.151 | –2.950 | 0.0044 |  | 0.300 |
| $\psi_{ij}=k\cdot\text{exp(}-\alpha VD_{ij}\text{)}\cdot N_{i}^{\zeta}\cdot T_{j}^{\xi}$ | 0.048 ± 0.018 | 2.748 | 0.0077 |  | 2.205 ± 0.449 | 4.914 | 0.0000 |  | –0.121 ± 0.117 | –1.033 | 0.3055 |  | –0.279 ± 0.110 | –2.540 | 0.0134 |  | 0.426 |
