# Supplementary material for: Sex-biased topography effects on butterfly dispersal
Source: Mov Ecol. 2020 Dec 14;8:50. doi: 10.1186/s40462-020-00234-6 (PMC7737334; doi:10.1186/s40462-020-00234-6)
Supplement: Supplementary file 4 — Additional file 4. Results - Model selection based on the Akaike InformationCriterion corrected for small sample size. [file 40462_2020_234_MOESM4_ESM.docx]

**Plazio E*, Bubová T, Vrabec V, Nowicki N (2020). Sex-biased topography effects on butterfly dispersal**

* Corresponding author. Email: [elisa.plazio@doctoral.uj.edu.pl](mailto:elisa.plazio@doctoral.uj.edu.pl)

**Additional file 4**. Model selection based on the Akaike Information Criterion corrected for small sample size. The predictors of movement probabilities of investigated *Maculinea* butterflies comprised *ED* = Euclidean distance, *VD* = valley distance, *N* = natal patch area, and *T* = target patch area. The most appropriate model according to the principle of parsimony, i.e. the one with the smallest number of parameters among the supported models with ΔAICc < 2, is presented in bold. It should be noted that several models, such as those with only patch areas but no inter-patch distance among predictors or a null model assuming constant movement probability, are included for comparison even if they were not thoroughly considered in our study and typically performed poorly.

| Model predictors | No. of parameters | –2log(Likelihood) | AICc | ΔAICc |
| --- | --- | --- | --- | --- |
| ***M. nausithous*, 2010, males** | | | | |
| *ED* and *T* | 3 | 325.188 | 331.541 | 0 |
| ***ED*** | **2** | **329.087** | **333.261** | **1.721** |
| *ED* and *N* and *T* | 4 | 325.185 | 333.782 | 2.242 |
| *ED* and *N* | 3 | 329.048 | 335.401 | 3.860 |
| *VD* and *T* | 3 | 329.054 | 335.407 | 3.866 |
| *VD* | 2 | 332.921 | 337.095 | 5.554 |
| *VD* and *N* and *T* | 4 | 329.044 | 337.641 | 6.101 |
| *VD* and *N* | 3 | 332.899 | 339.252 | 7.711 |
| *T* | 2 | 347.984 | 352.158 | 20.618 |
| constant | 1 | 351.340 | 353.398 | 21.857 |
| *N* and *T* | 3 | 347.942 | 354.295 | 22.755 |
| *N* | 2 | 351.340 | 355.514 | 23.973 |
| ***M. nausithous*, 2010, females** | | | | |
| ***VD*** | **2** | **309.788** | **313.962** | **0** |
| *VD* and *N* | 3 | 309.614 | 315.967 | 2.005 |
| *VD* and *T* | 3 | 309.788 | 316.140 | 2.179 |
| *ED* | 2 | 312.918 | 317.092 | 3.130 |
| *VD* and *N* and *T* | 4 | 309.610 | 318.207 | 4.245 |
| *ED* and *N* | 3 | 312.730 | 319.083 | 5.122 |
| *ED* and *T* | 3 | 312.917 | 319.270 | 5.308 |
| *ED* and *N* and *T* | 4 | 312.722 | 321.319 | 7.358 |
| constant | 1 | 333.182 | 335.240 | 21.278 |
| *N* | 2 | 333.125 | 337.299 | 23.338 |
| *T* | 2 | 333.171 | 337.345 | 23.383 |
| *N* and *T* | 3 | 333.119 | 339.472 | 25.511 |

**Additional file 4**. *continued*

| Model predictors | No. of parameters | –2log(Likelihood) | AICc | ΔAICc |
| --- | --- | --- | --- | --- |
| ***M. nausithous*, 2014, males** | | | | |
| *ED* and *T* | 3 | 323.378 | 329.731 | 0 |
| ***ED*** | **2** | **326.238** | **330.412** | **0.681** |
| *ED* and *N* and *T* | 4 | 322.171 | 330.768 | 1.037 |
| *ED* and *N* | 3 | 325.729 | 332.082 | 2.351 |
| *VD* and *T* | 3 | 327.207 | 333.560 | 3.829 |
| *VD* and *N* and *T* | 4 | 325.954 | 334.551 | 4.819 |
| *VD* | 2 | 333.941 | 338.115 | 8.384 |
| *VD* and *N* | 3 | 333.396 | 339.749 | 10.018 |
| *T* | 2 | 346.040 | 350.214 | 20.483 |
| *N* and *T* | 3 | 344.827 | 351.180 | 21.449 |
| constant | 1 | 351.747 | 353.804 | 24.072 |
| *N* | 2 | 351.177 | 355.351 | 25.620 |
| ***M. nausithous*, 2014, females** | | | | |
| *VD* and *N* | 3 | 324.647 | 331.000 | 0 |
| ***VD*** | **2** | **327.157** | **331.331** | **0.332** |
| *VD* and *N* and *T* | 4 | 324.591 | 333.188 | 2.188 |
| *VD* and *T* | 3 | 327.156 | 333.509 | 2.510 |
| *ED* | 2 | 338.157 | 342.331 | 11.331 |
| *ED* and *N* | 3 | 335.990 | 342.343 | 11.343 |
| *ED* and *T* | 3 | 338.156 | 344.508 | 13.509 |
| *ED* and *N* and *T* | 4 | 335.939 | 344.536 | 13.537 |
| *N* | 2 | 348.690 | 352.864 | 21.864 |
| constant | 1 | 350.819 | 352.876 | 21.876 |
| *N* and *T* | 3 | 348.580 | 354.933 | 23.933 |
| *T* | 2 | 350.798 | 354.972 | 23.972 |

**Additional file 4**. *continued*

| Model predictors | No. of parameters | –2log(Likelihood) | AICc | ΔAICc |
| --- | --- | --- | --- | --- |
| ***M. teleius*, 2010, males** | | | | |
| ***ED*** | **2** | **327.975** | **332.149** | **0** |
| *VD* | 2 | 329.745 | 333.919 | 1.770 |
| *ED* and *T* | 3 | 327.702 | 334.055 | 1.906 |
| *ED* and *N* | 3 | 327.938 | 334.291 | 2.142 |
| *VD* and *T* | 3 | 329.447 | 335.800 | 3.651 |
| *VD* and *N* | 3 | 329.719 | 336.072 | 3.923 |
| *ED* and *N* and *T* | 4 | 327.686 | 336.283 | 4.134 |
| *VD* and *N* and *T* | 4 | 329.438 | 338.035 | 5.886 |
| constant | 1 | 338.932 | 340.989 | 8.840 |
| *T* | 2 | 338.586 | 342.760 | 10.611 |
| *N* | 2 | 338.926 | 343.100 | 10.951 |
| *N* and *T* | 3 | 338.586 | 344.939 | 12.790 |
| ***M. teleius*, 2010, females** | | | | |
| *VD* and *N* and *T* | 4 | 323.077 | 331.674 | 0 |
| *VD* and *T* | 3 | 325.876 | 332.229 | 0.555 |
| ***VD*** | **2** | **328.551** | **332.725** | **1.051** |
| *VD* and *N* | 3 | 328.241 | 334.594 | 2.920 |
| *ED* and *T* | 3 | 330.953 | 337.306 | 5.631 |
| *ED* and *N* and *T* | 4 | 330.221 | 338.818 | 7.144 |
| *ED* | 2 | 336.084 | 340.258 | 8.583 |
| *ED* and *N* | 3 | 335.796 | 342.149 | 10.475 |
| *T* | 2 | 338.811 | 342.985 | 11.310 |
| *N* and *T* | 3 | 337.981 | 344.334 | 12.660 |
| constant | 1 | 343.803 | 345.860 | 14.186 |
| *N* | 2 | 343.447 | 347.621 | 15.947 |

**Additional file 4**. *continued*

| Model predictors | No. of parameters | –2log(Likelihood) | AICc | ΔAICc |
| --- | --- | --- | --- | --- |
| ***M. teleius*, 2014, males** | | | | |
| ***ED*** | **2** | **346.520** | **350.694** | **0** |
| *ED* and *T* | 3 | 346.266 | 352.619 | 1.925 |
| *ED* and *N* | 3 | 346.381 | 352.734 | 2.039 |
| *VD* | 2 | 348.982 | 353.156 | 2.462 |
| *ED* and *N* and *T* | 4 | 346.071 | 354.668 | 3.974 |
| *VD* and *T* | 3 | 348.703 | 355.056 | 4.362 |
| *VD* and *N* | 3 | 348.822 | 355.175 | 4.481 |
| *VD* and *N* and *T* | 4 | 348.481 | 357.078 | 6.384 |
| constant | 1 | 357.792 | 359.849 | 9.154 |
| *T* | 2 | 357.465 | 361.639 | 10.945 |
| *N* | 2 | 357.589 | 361.762 | 11.068 |
| *N* and *T* | 3 | 357.187 | 363.540 | 12.845 |
| ***M. teleius*, 2014, females** | | | | |
| *VD* and *N* | 3 | 331.031 | 337.384 | 0 |
| ***VD*** | **2** | **333.289** | **337.463** | **0.079** |
| *VD* and *T* | 3 | 332.978 | 339.331 | 1.946 |
| *VD* and *N* and *T* | 4 | 330.888 | 339.485 | 2.101 |
| *ED* | 2 | 339.373 | 343.547 | 6.163 |
| *ED* and *N* | 3 | 337.292 | 343.645 | 6.261 |
| *ED* and *T* | 3 | 339.090 | 345.443 | 8.058 |
| *ED* and *N* and *T* | 4 | 337.163 | 345.760 | 8.376 |
| constant | 1 | 347.438 | 349.495 | 12.111 |
| *N* | 2 | 345.324 | 349.498 | 12.114 |
| *T* | 2 | 347.268 | 351.442 | 14.057 |
| *N* and *T* | 3 | 345.268 | 351.621 | 14.236 |
